# Diagnostic Accuracy of the Cobas^®^ MTB and Cobas MTB/RIF-INH Assays on Sputum and the Cobas MTB Assay on Tongue Swabs for *Mycobacterium tuberculosis* Complex Detection in Symptomatic Adults in South Africa

**DOI:** 10.3390/biomedicines13102556

**Published:** 2025-10-20

**Authors:** Anura David, Lyndel Singh, Manuel Pedro da Silva, Keneilwe Peloakgosi-Shikwambani, Zanele Nsingwane, Violet Molepo, Wendy Stevens, Lesley Erica Scott

**Affiliations:** 1 Wits Diagnostic Innovation Hub, Health Sciences Research Office, Faculty of Health Sciences, University of the Witwatersrand, Johannesburg 2193, South Africa; lyndel.singh@witsdih.ac.za (L.S.); pkeneilwe@yahoo.com (K.P.-S.); zanele.nsingwane@witsdih.ac.za (Z.N.); violet.molepo@witsdih.ac.za (V.M.); wendy.stevens@wits.ac.za (W.S.); lesley.scott@wits.ac.za (L.E.S.); 2 National Priority Program, National Health Laboratory Service, Johannesburg 2193, South Africa; pedro.dasilva@nhls.ac.za

**Keywords:** nucleic acid amplification test, Cobas MTB, *Mycobacterium tuberculosis* complex, drug resistance, rifampicin, isoniazid

## Abstract

**Background/Objectives**: Accurate and rapid detection of *Mycobacterium tuberculosis* complex (MTBC) and drug resistance is essential for effective tuberculosis (TB) management, particularly in high-burden settings. The Cobas^®^ MTB and Cobas MTB/RIF-INH assays are moderate-complexity nucleic acid amplification tests that detect MTBC and resistance to rifampicin (RIF) and isoniazid (INH). **Methods**: This study evaluated the clinical diagnostic performance of the Cobas assays on sputum, using liquid culture as the reference standard and Xpert MTB/RIF Ultra (Xpert Ultra) for comparison. Diagnostic accuracy of the Cobas MTB assay on tongue swabs (TS) was also assessed. **Results**: In a study population (*n* = 354) with 56% HIV prevalence, the overall sensitivity and specificity of Cobas MTB on sputum was 93.8% (95% CI: 84.8–98.3) and 100% (95% CI: 98.7–100) compared with culture. The assay showed almost perfect agreement with Xpert Ultra (Cohen’s kappa = 0.904). Among HIV-positive participants, sensitivity was 88.2% (95% CI: 72.5–96.7). RIF resistance profiling by Cobas MTB/RIF-INH was fully concordant with culture and Xpert Ultra. Three INH-resistant cases were missed, likely due to genotypic–phenotypic discordance. Although specimen numbers were small, TS demonstrated better diagnostic accuracy when using a diluted (66%) microbial inactivation solution. **Conclusions**: The Cobas MTB and MTB/RIF-INH assays demonstrated high diagnostic accuracy compared to culture and Xpert Ultra on sputum. Findings support TS as an alternative specimen type for MTBC detection using an optimized protocol. These findings underscore the potential of the Cobas assays as reliable alternatives for TB and resistance diagnostics, particularly in settings where rapid, accurate detection of MTBC and RIF or INH resistance is crucial.

## 1. Introduction

The Cobas^®^ 6800/8800 systems (Roche Molecular Systems Inc., Pleasanton, CA, USA) have been integral to South Africa’s diagnostic landscape. These high-throughput platforms were introduced in 2015 [1] for the testing of the Cobas HIV-1 quantitative nucleic acid amplification test (NAAT) and have since played a critical role in HIV management in a country with one of the largest populations of people living with HIV (PLHIV). The Cobas systems have additionally demonstrated versatility by supporting alternative specimen types, such as plasma separation cards [2], thereby providing an option for testing conventional plasma samples.

It is not surprising that a country with such high HIV rates also ranks as one of the countries with the highest burden of tuberculosis (TB) and multi-drug-resistant TB (MDR-TB) globally [3]. For TB diagnosis, the Xpert MTB/RIF [Xpert] (Cepheid, Sunnyvale, CA, USA) assay has been used as the initial diagnostic since 2011, followed by the Xpert MTB/RIF Ultra (Xpert Ultra) assay in 2017. The shortage of Xpert Ultra cartridges experienced during (and after) the COVID-19 pandemic highlighted the risks of reliance on a single supplier. This shortage, together with the growing diagnostic pipeline [4], as well as the WHO recommendation for the use of a new category of diagnostics, the moderate-complexity assays, in 2021 [5], led to the diversification of the molecular platforms used in the South African National TB program [6]. Since 2023, alongside the Xpert Ultra assay, the Cobas MTB and MTB RIF/INH assays (Roche Molecular Systems Inc., Pleasanton, CA, USA) and the MAX MDR-TB (Becton, Dickinson and Company, Sparks, MD, USA) assays have been incorporated into the TB testing algorithm. Since the Cobas systems were already in use for the HIV program with established in-country support, integrating a test utilizing the same technology provided a strategic advantage.

The Cobas MTB assay is an automated, qualitative, real-time polymerase chain reaction (PCR) test designed to detect *Mycobacterium tuberculosis* complex (MTBC) DNA in respiratory specimens. If an MTBC-positive result is detected on the assay, the specimen can be reflexed to the Cobas MTB/RIF-INH assay, which detects rifampicin (RIF) resistance-associated mutations of the *rpoB* gene, and isoniazid (INH) resistance-associated mutations in the *katG* and *inhA* genes. Both assays were previously evaluated by our group, first in an analytical evaluation using spiked sputum specimens and culture isolates [7] and then in a multi-country clinical performance evaluation [8]. However, the clinical evaluation study design excluded individuals unable to produce ≥2 mL of sputum, effectively omitting those who typically present for TB investigation in South Africa (SA) with sputum volumes of around 1 mL. We previously evaluated the Cobas MTB assay in SA only [9], but the comparator used in that study was Xpert, not the currently used Xpert Ultra.

Other studies have assessed the performance of the Cobas TB assays using N-acetyl-l-cysteine-sodium hydroxide (NALC-NaOH)-treated specimens [10] or culture isolates and spiked sputum [11], but data on raw sputum is limited.

In this clinical performance evaluation on sputum, we assessed the performance of the Cobas MTB and Cobas MTB-RIF/INH assays for the detection of MTBC, RIF, and INH resistance compared to a liquid culture reference standard and to Xpert Ultra as a comparator. Given our group’s focus on exploring tongue swabs (TSs) as an additional specimen type for TB diagnosis, we collected TS in parallel with sputum and evaluated the diagnostic accuracy of the Cobas MTB assay for MTBC detection in TS specimens.

## 2. Materials and Methods

### 2.1. Study Design and Procedures

We conducted a cross-sectional, prospective study to assess the accuracy of the Cobas MTB assay on sputum, using the mycobacterial growth indicator tube (MGIT) (Becton Dickinson, Sparks, MD, USA) liquid culture as the reference standard. The performance of the Cobas MTB assay was also compared with that of the Xpert Ultra assay. Additionally, we evaluated the concordance of the Cobas MTB assay on TSs against Xpert Ultra, liquid culture, and Cobas MTB results from sputum.

**Figure 1:**
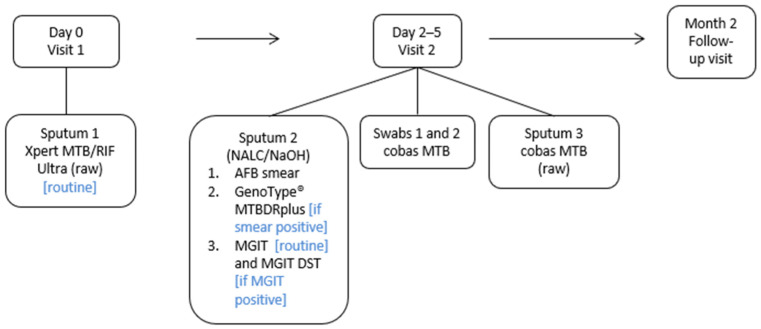
Description of study outline indicating clinic visits and specimen laboratory pathways.

Symptomatic adults (≥18 years) attending the Hillbrow Community Health Centre (HCHC) in Johannesburg, Gauteng, SA, being investigated for TB were approached and invited to enroll in the study. Recruitment occurred from 20 October 2021 to 11 July 2023. The research nurse performed the WHO-recommended four-symptom screen (W4SS)—cough, fever, weight loss, and night sweats. Personal characteristics such as age, weight, height, HIV status, and previous TB history were also recorded. Weight and height measurements were used to calculate the body mass index (BMI), which was used as an indicator of nutritional status. Participants with a BMI below 18.5 kg/m^2^ were classified as underweight, suggesting possible malnutrition. Study inclusion criteria included participant willingness to return for a second visit, provision of the required number of study specimens, and absence of any TB treatment within six months prior to enrolment. Specimen collection was performed for routine and research testing over two visits (Figure 1). Research nurses used two Copan FLOQSwab (Copan, Brescia, Italy) swabs for tongue collection from each participant. Collection was performed by swabbing the dorsum of the tongue for 30 s as far back on the tongue as possible without initiating a gag reflex. One TS was collected before sputum collection and the second after sputum collection. Participants abstained from consuming any food or beverages for at least 30 min prior to specimen collection. Both swabs were transported “dry” (without any transport buffer) to the laboratory for testing.

Routine and research testing was performed at the Wits Diagnostic Innovation Hub (WitsDIH) research laboratory in Braamfontein (Johannesburg).

Specimens for routine and study testing were collected over two visits approximately 2–5 days apart. Arrows indicate the chronological flow of study visits and specimen collection. Day 0 (Visit 1) procedures were followed by Day 2–5 (Visit 2), and subsequently by the Month 2 follow-up visit. Blue text in brackets indicates conditions under which specific tests were performed. AFB, acid-fast bacilli; MGIT, mycobacterial growth indicator tube; DST, drug susceptibility testing; NALC/NaOH, N-acetyl-L-cysteine–sodium citrate–sodium hydroxide; BD, Becton Dickinson

### 2.2. Laboratory Testing

Genotype^®^ MTBDR*plus* line probe assay (LPA) (Hain Lifescience/Bruker, Nehren, Germany) testing was performed on all smear-positive sputum and also used to speciate liquid cultures that were acid-fast bacilli (AFB)-positive. Routine testing and result reporting was performed by laboratory staff in accordance with the National Tuberculosis Management Guidelines [12]. Staff performing routine testing were blinded to Cobas MTB results.

Cobas MTB testing on sputum was performed according to manufacturer instructions. Sputum was stored at −20 °C until batch testing. Microbial inactivation solution (MIS) (Roche Molecular Systems Inc., Pleasanton, CA, USA) was added to raw sputum at a 2:1 ratio of solution to specimen. This mix was incubated at room temperature for 60 min, followed by sonication and a 5 min centrifugation. Testing was performed on the Cobas 6800 System. Results are reported as MTB-positive, MTB-negative, or invalid (test was unable to produce a reliable or interpretable result due to issues with the specimen or the testing process). Any specimen which produced an MTB-positive result was reflexed for RIF and INH testing using the Cobas MTB/RIF-INH assay. Research staff performed Cobas testing and were blinded to routine TB results.

Swabs were stored at −80 °C until batch testing. For processing of TSs, 1.8 mL of MIS was added to the “dry” swab and 1.5 mL was loaded on the Cobas 6800 System. Processing of TSs was performed according to the standard sputum protocol using neat MIS, with the exception that the centrifugation step was excluded (*n* = 239). However, parallel testing on spiked swabs showed improved sensitivity using a diluted (66%) MIS buffer; hence, 66% MIS buffer was used to process the remaining swabs (*n* = 99).

### 2.3. Outcomes and Statistical Analysis

For diagnostic accuracy, results from the Cobas MTB assay on sputum were compared with liquid culture for MTBC detection. Additionally, resistance profiling for RIF and INH was evaluated by comparing Cobas MTB assay results to phenotypic drug susceptibility testing (pDST) using the MGIT960 SIRE kit (streptomycin [S], isoniazid [I], rifampicin [R], and ethambutol [E]; Becton Dickinson, Sparks, MD, USA). Data analysis included calculation of sensitivity, specificity, positive predictive value (PPV), and negative predictive value (NPV) for MTBC detection. All metrics were reported with 95% confidence intervals (CIs), calculated using the Wilson score method. Analyses were performed both for all participants and after excluding those with an Ultra “trace” result. Cobas MTB results were additionally compared with Xpert Ultra results. For Cobas MTB assay performance on TS, concordance with Xpert Ultra, liquid culture, and Cobas MTB sputum is reported.

The target sample size was calculated using the following formula:

$$n\;=\;\frac{Z^{2}Xp(1-p)}{x^{2}}$$

where *n* is the required sample size, *Z* is the standard normal deviate corresponding to the desired confidence level (1.96 for 95% confidence), *p* is the estimated population proportion, and *x* is the desired precision (expressed as a proportion). We selected *p* = 0.50 as a conservative estimate, as this maximizes the product *p*(1 − *p*) when the true proportion is unknown, and *x* = 0.05 to achieve a 95% confidence interval with ±5% precision. Substituting these values into the formula yields *n* ≈ 384. To allow for attrition and participants who did not meet the inclusion criteria, the target sample size was rounded up to 400 participants.

To determine the performance of the Cobas MTB assay, only specimens that generated valid results across all tests (Cobas MTB, Ultra and liquid culture) were included in the statistical analysis.

## 3. Results

### 3.1. Characteristics of the Study Population

**Figure 2:**
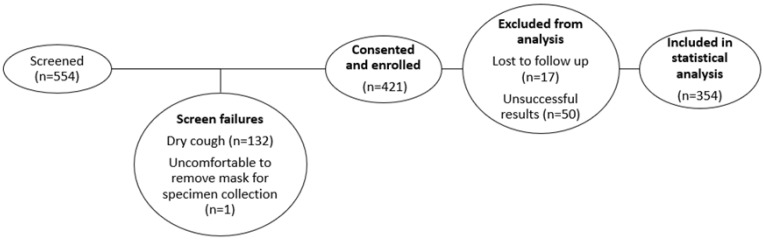
Data description for statistical analysis.

A total of 554 participants were screened for the study (Figure 2). Of these, 133 were excluded during the recruitment process, and 421 participants gave consent and were enrolled. Of those enrolled, 67 participants were excluded, yielding a final sample of 354 participants for statistical analysis. Characteristics of the study population are shown in Table 1. The average age of participants was 39 years, and most (64%) were male. As indicated by their BMI, a total of 28/66 (42%) participants diagnosed with active TB were malnourished. Among participants with a known HIV status, 57% (199/351) were positive. Forty-two reported a previous TB episode. TB was microbiologically confirmed on liquid culture in 64/354 (18%) of participants. A total of 290 participants did not have microbiologically confirmed TB and were consequently classified as not having active TB disease. The culture contamination rate was 11% (43/404); of these, the Ultra and Cobas MTB assays detected one MTBC-positive specimen, and the participant reported an improvement in symptoms after having received treatment. Sixty-six participants were microbiologically confirmed to have TB using the Xpert Ultra assay.

### 3.2. Clinical Performance Evaluation of the Cobas MTB Assay on Sputum

The performance of the Cobas MTB assay on raw sputum was compared with the reference method of liquid culture and, additionally, stratified by HIV and smear status, as outlined in Table 2 for 354 participant results. The assay exhibited high sensitivity (94%) and 100% specificity, with improved performance observed when Xpert Ultra “trace” results were excluded. Assay sensitivity among HIV-positive individuals was 88%, while perfect sensitivity (100%) and specificity (100%) was achieved in HIV-negative individuals. The Cobas MTB assay produced 1/404 (<1%) invalid results. Unsuccessful tests were not repeated due to cost considerations.

### 3.3. Comparison of the Cobas MTB Assay to Xpert Ultra for Detection of MTBC

The Cobas MTB and Xpert Ultra assays demonstrated a high level of concordance, with an overall agreement of 97.5% (344/354) and a Cohen’s kappa coefficient of 0.904 (95% CI: 0.8447–0.9623).

Among the discordant results, eight specimens were positive by Xpert Ultra but negative by Cobas MTB; all eight had “trace” or “very low” semi-quantitative results on Ultra, and two of these were culture positive. Conversely, two specimens were positive by Cobas MTB but negative by Xpert Ultra; both were also confirmed positive by liquid culture.

### 3.4. Clinical Performance Evaluation of the Cobas MTB/RIF-INH Assay on Sputum

Of the 60 raw sputum which produced an MTBC-positive result on the Cobas MTB assay and were reflex-tested on the Cobas MTB/RIF-INH assay, reportable resistance results were available for 52 (87%) specimens. Unsuccessful tests were not repeated due to insufficient specimen volumes.

For RIF resistance detection, the Cobas MTB/RIF-INH assay accurately identified correct resistance profiles in 47/47 (100%) sputum samples with valid results (Table 3). Of the specimens tested, 8/60 (13%) yielded invalid results. Where pDST was unsuccessful, the assay successfully assigned resistance profiles to five specimens, as confirmed by the LPA.

For INH resistance detection, the Cobas MTB/RIF-INH assay correctly identified resistance profiles in 44/47 (94%) sputum samples with valid results (Table 4). In three cases, the assay did not detect INH resistance that was identified by pDST, with resistance also undetected by LPA in 1/3 (33%) of these cases. Additionally, 8/60 (13%) specimens produced invalid results; however, the assay successfully assigned resistance profiles to five specimens, as confirmed by LPA, where pDST was unsuccessful.

### 3.5. Diagnostic Performance of Tongue Swabs on the Cobas MTB Assay

When compared to Xpert Ultra, TSs detected MTBC across all semi-quantitative categories, from “high” to “very low.” Using undiluted MIS, MTBC detection was observed in three participants on TSs collected after sputum, compared to those collected before (Table 5). However, with 66% MIS, overall detection improved with comparable detection rates between TSs collected before and after sputum. Similarly, when compared to liquid culture or Cobas MTB sputum and processed with 66% MIS, TSs showed improved MTBC detection and comparable detection rates on Cobas MTB. Regardless of the comparator used, assay specificity remained consistent between undiluted and 66% MIS.

## 4. Discussion

This study investigated the clinical performance of the Cobas MTB and Cobas MTB/RIF-INH assays for MTBC, RIF, and INH resistance detection using raw sputum against a liquid culture reference standard and Xpert Ultra as a comparator. In addition, we investigated the diagnostic accuracy of the Cobas MTB assay on TSs compared with Xpert Ultra sputum, liquid culture, and Cobas MTB sputum.

In this study population with an HIV prevalence of 57%, the Cobas MTB assay demonstrated high sensitivity (94%) relative to culture, comparable to the Xpert Ultra assay (94%), and achieved a specificity of 100%. Among HIV-positive participants, the Cobas MTB assay showed a sensitivity of 88%, compared to 91% for Xpert Ultra. In this subset, when participants with “trace” semi-quantitative Xpert Ultra results were excluded from the analysis, the sensitivity of the Cobas MTB assay improved to 97%. The assay exhibited perfect sensitivity (100%) within the HIV-negative subset of participants. There was almost perfect agreement [13] between the Cobas MTB and Xpert Ultra assays, reflecting a high degree of concordance, with both assays demonstrating similar unsuccessful rates of <1%. Thus, in terms of performance, both assays perform comparably, which supports use of the Cobas MTB assay in South Africa’s TB testing diversification.

Xpert Ultra has the advantage of providing an MTBC result and an upfront RIF resistance profile within a shorter period and fewer pre-processing steps compared to the Cobas MTB assay [14]. However, the Cobas assay, despite requiring reflex resistance testing, can provide an INH result, which Xpert Ultra cannot. This has implications for patient management, as a South African prevalence survey (2012–2014) found that INH-monoresistant TB accounted for more than 5% of cases in all provinces [15]. Since testing is performed on the high-throughput 68/8800 systems, the Cobas MTB assay is suited for high-burden settings or in countries which are trying to ramp up TB screening in attempts to overcome the effects of COVID-19 [16]. An additional advantage of the Cobas MTB assay is that resistance testing can be performed only when required, which will save costs. When compared to other WHO-endorsed moderate-complexity assays, the Cobas MTB assay demonstrated similar analytical performance [7] to Xpert and, in terms of ease of use and operational requirements, achieved the second-highest score, after the MAX MDR-TB assay [14].

For RIF resistance detection, the Cobas MTB/RIF-INH assay showed perfect agreement with liquid culture and Xpert Ultra. Both Xpert Ultra and Cobas MTB/RIF-INH yielded an unsuccessful rate of 13% for RIF detection. For INH, resistance was missed in three specimens (as per the pDST result), one of which was also missed by the LPA. One explanation for this is that it has been shown that phenotypic resistance may sometimes be identified before the associated genetic mutations can be detected since molecular assays require a higher drug-resistant proportion to be present. This mismatch can arise from the limited sensitivity of genotypic assays, which generally focus on the most frequently occurring mutations, or from resistance mechanisms that have not yet been genetically characterized [17].

Tongue swabs are increasingly explored as a non-invasive additional specimen type to sputum for MTBC detection, particularly for individuals who cannot expectorate, and offer advantages in patient acceptability and ease of collection [18]. However, their sensitivity remains variable, ranging from approximately 36% to 91% in adults, largely due to a limitation in the number of bacilli captured on the swab, differences in swab types, collection methods, and pre-analytical processing [18].

For the Cobas MTB assay on TSs, MTBC detection was comparable between TSs collected before and after sputum collection, suggesting that the timing of collection does not affect assay performance. When evaluated against Xpert Ultra sputum semi-quantitative results, the Cobas MTB assay on TSs detected MTBC down to the “very low” category, with improved detection in participants with higher bacterial loads, though some variability was observed. These findings align with those reported by Ahls et al. [19], further supporting the idea that MTBC detection using TSs improves with increasing bacterial load. Cobas MTB assay performance on sputum was better than that seen on TSs, but this is expected since the assay is designed for sputum, and additional specimen types will likely require protocol modification. This study also explored protocol modifications by testing a diluted MIS (66%) on a subset of specimens to assess whether assay sensitivity could be improved. Undiluted MIS is necessary for sputum processing to effectively liquefy and inactivate the dense, mucopurulent material; however, this same concentration may be too harsh for tongue swabs, potentially reducing the recovery of intact MTBC DNA. A reduced MIS concentration may preserve more detectable DNA, thereby improving sensitivity. While the number of MTBC-positive specimens in this study was too limited to draw definitive conclusions, assay sensitivity and specificity seemed improved using 66% MIS. Future studies should consider this diluted buffer for TS pre-processing for testing on the Cobas MTB assay.

## 5. Conclusions

In conclusion, the Cobas MTB and Cobas MTB/RIF-INH assays demonstrated comparable performance to Xpert Ultra on sputum when compared to liquid culture, showcasing high sensitivity and specificity for MTBC detection. These findings highlight the potential of the Cobas assays as reliable alternatives for TB diagnosis, particularly in settings where rapid, high-throughput testing is needed. While the assays also detect RIF and INH resistance, the limited number of resistant specimens in this study constrains the strength of conclusions regarding resistance detection performance. The study findings further support the potential utility of TSs as an additional specimen type for MTBC detection on the Cobas MTB assay, particularly when using an optimized pre-processing protocol such as diluted (66%) MIS; however, this observation is based on a small subset of specimens and requires validation in larger cohorts. Future studies should investigate the application of Cobas assays in diverse patient populations and operational contexts to better define their role in global TB control efforts.

## Figures and Tables

**Table 1:** Cohort characteristics for participants used in the statistical analysis.

Characteristic	All (*n* = 354)
Demographics	
Age, mean (range), years	39 (18–70)
Male sex, *n* (%)	213 (63.6)
BMI kg/m^2^, *n* (%)	
<18.5 (Underweight)	94 (26.6)
18.5–24.9 (Healthy weight)	186 (52.5)
25–29.9 (Overweight)	44 (12.4)
>30 (Obese)	30 (8.5)
HIV-related information	
HIV-positive, *n* (%)	199 (56.6)
HIV-negative, *n* (%)	152 (42.9)
Status unknown, *n* (%)	3 (0.9)
TB History	
Previously diagnosed with TB, *n* (%)	42 (11.9)
Clinical signs and symptoms of TB at presentation	
Cough (any duration), *n* (%)	353 (99.7)
Unexplained weight loss, *n* (%)	265 (74.9)
Nights sweats, *n* (%)	263 (74.2)
Fever, *n* (%)	236 (66.9)
* Other, *n* (%)	257 (72.6)
Bacteriological confirmation (sputum 2), *n* (%)	
Smear and culture positive	44 (12.4)
Smear negative and culture positive	20 (5.6)
Smear and culture negative	284 (80.2)
Smear positive and culture negative	6 (1.7)

BMI, body mass index. * Other symptoms include body aches, loss of appetite, hemoptysis, shortness of breath, dizziness, and vomiting.

**Table 2:** The performance of smear microscopy, Xpert Ultra, and Cobas MTB assays compared to the reference standard (liquid culture) for MTBC detection on sputum.

Variable	Smear Microscopy	Xpert MTB/RIF Ultra (Raw Sputum)	Cobas MTB (Raw Sputum)
All (including trace) (*n* = 354)			
Sensitivity, % (95% CI)	68.8 (55.9–79.8)	93.8 (84.8–98.3)	93.8 (84.8–98.3)
Specificity, % (95% CI)	97.9 (95.6–99.2)	97.9 (95.6–99.2)	100 (98.7–100)
PPV, % (95% CI)	88.2 (76.1–95.6)	90.9 (81.3–96.6)	100 (94.0–100)
NPV, % (95% CI)	93.4 (90.0–95.9)	98.6 (96.5–99.6)	98.6 (96.6–99.6)
All (excluding trace) (*n* = 347)			
Sensitivity, % (95% CI)	70.5 (57.4–81.5)	93.4 (84.1–98.2)	96.7 (88.7–99.6)
Specificity, % (95% CI)	97.9 (95.5–99.2)	99.3 (97.5–99.9)	100 (98.7–100)
PPV, % (95% CI)	87.8 (75.2–95.4)	96.6 (88.3–99.6)	100 (93.9–100)
NPV, % (95% CI)	94.0 (90.6–96.4)	98.6 (96.5–99.6)	99.3 (97.5–99.9)
Specimens from HIV-positive individuals (*n* = 199)			
Sensitivity, % (95% CI)	58.8 (40.7–75.4)	91.2 (76.3–98.1)	88.2 (72.5–96.7)
Specificity, % (95% CI)	98.2 (94.8–99.6)	97.6 (93.9–99.3)	100 (97.8–100)
PPV, % (95% CI)	87.0 (66.4–97.2)	88.6 (73.3–96.8)	100 (88.4–100)
NPV, % (95% CI)	92.0 (87.0–95.6)	98.2 (94.7–99.6)	97.6 (94.1–99.4)
Specimens from HIV-negative individuals (*n* = 152)			
Sensitivity, % (95% CI)	82.1 (63.1–93.9)	96.4 (81.7–99.9)	100 (87.7–100)
Specificity, % (95% CI)	97.6 (93.1–99.5)	98.4 (94.0–99.8)	100 (97.1–100)
PPV, % (95% CI)	88.5 (69.8–97.6)	93.1 (77.2–99.2)	100 (87.7–100)
NPV, % (95% CI)	96.0 (91.0–98.7)	99.1 (95.3–100)	100 (97.1–100)
Smear microscopy negative specimens (*n* = 304)			
Sensitivity, % (95% CI)	*n*/a	85.0 (62.1–96.8)	85.0 (62.1–96.8)
Specificity, % (95% CI)		97.9 (95.5–99.2)	100 (98.7–100)
PPV, % (95% CI)		73.9 (51.6–89.8)	100 (80.5–100)
NPV, % (95% CI)		98.9 (96.9–99.8)	99.0 (97.0–99.8)

PPV, positive predictive value; NPV, negative predictive value; CI, confidence interval.

**Table 3:** Cobas RIF resistance results, compared to phenotypic DST.

pDST Result	Cobas MTB-RIF/INH Result		
	RIF-R not detected	RIF-R detected	Invalid
RIF-sensitive (*n* = 54)	46	0	8
RIF-resistant (*n* = 1)	0	1	0
Invalid (*n* = 5)	5	0	0

RIF, rifampicin; RIF-R, rifampicin resistance; pDST, phenotypic drug susceptibility testing.

**Table 4:** Cobas INH resistance results compared to phenotypic DST.

pDST Result	Cobas MTB-RIF/INH Result		
	INH-R not detected	INH-R detected	Invalid
INH sensitive (*n* = 50)	43	0	7
INH resistant (*n* = 5)	3	1	1
Invalid (*n* = 5)	5	0	0

INH, isoniazid; INH-R, isoniazid resistance; pDST, phenotypic drug susceptibility testing.

**Table 5:** Performance of the Cobas MTB assay on tongue swabs compared to Xpert Ultra, liquid culture, and Cobas MTB sputum results.

	Cobas MTB TS Result, *n*/*N* (%)			
Comparator Test Result (Sputum)	Neat MIS (TS 1)	Neat MIS (TS 2)	66% MIS (TS 1)	66% MIS (TS 2)
Xpert Ultra-positive	25/53 (47.2)	28/53 (52.8)	9/13 (69.2)	9/13 (69.2)
High	16/18 (88.9)	15/18 (83.3)	4/6 (66.7)	4/6 (66.7)
Medium	2/6 (33.3)	3/6 (50.0)	1/2 (50.0)	1/2 (50.0)
Low	6/15 (40.0)	6/15 (40.0)	4/4 (100)	4/4 (100)
Very low	1/7 (14.2)	4/7 (57.1)	0/1 (0)	0/1 (0)
Trace	0/7 (0)	0/6 (0)	-	-
Xpert Ultra-negative	215/217 (99.1)	216/217 (99.5)	70/71 (98.6)	71/71 (100)
Liquid culture-positive	26/52 (50)	29/52 (55.8)	9/12 (75.0)	9/12 (75.0)
Liquid culture-negative	217/218 (99.5)	218/218 (100)	71/72 (98.6)	72/72 (100)
Cobas MTB sputum-positive	26/48 (54.2)	29/48 (60.4)	9/12 (75.0)	9/12 (75.0)
Cobas sputum-negative	221/222 (99.5)	222/222 (100)	71/72 (98.6)	72/72 (100)

MIS, microbial inactivation solution; TS, tongue swab; TS 1 was collected before sputum collection and TS 2 after sputum collection.

## Data Availability

The protocol and raw data supporting the conclusions of this article will be made available by the authors on request.

## Abbreviations

The following abbreviations are used in this manuscript:

AFB	Acid fast bacilli
BD	Becton Dickinson
BMI	Body mass index
CI	Confidence interval
DNA	Deoxyribonucleic acid
DST	Drug susceptibility testing
HCHC	Hillbrow Community Health Centre
INH	Isoniazid
LPA	Line probe assay
MDR-TB	Multi-drug-resistant TB
MGIT	Mycobacterial growth inidicator tube
MIS	Microbial inactivation solution
MTBC	*Mycobacterium tuberculosis* complex
NAAT	Nucleic acid amplification test
NALC-NaOH	N-acetyl-l-cysteine-sodium hydroxide
NPV	Negative predictive value
PCR	Polymerase chain reaction
pDST	Phenotypic drug susceptibility testing
PLHIV	People living with HIV
PPV	Positive predictive value
SIRE	Streptomycin [S], isoniazid [I], rifampicin [R], and ethambutol [E]
SR	Sample reagent
RIF	Rifampicin
TB	Tuberculosis
TS	Tongue swab
W4SS	WHO-recommended four-symptom screen
WitsDIH	Wits Diagnostic Innovation Hub
